# Effect of Saturated Steam Heat Treatment on Physical and Chemical Properties of Bamboo

**DOI:** 10.3390/molecules25081999

**Published:** 2020-04-24

**Authors:** Qiuyi Wang, Xinwu Wu, Chenglong Yuan, Zhichao Lou, Yanjun Li

**Affiliations:** 1 College of Materials Science and Engineering, Nanjing Forestry University, Nanjing 210037, China; lethe813@163.com (Q.W.); Xinwu_Wu2018@163.com (X.W.); Chenglong_Yuan2018@163.com (C.Y.); 2 Jiangsu Co-Innovation Center of Efficient Processing and Utilization of Forest Resources, Nanjing 210037, China

**Keywords:** bamboo, saturated steam heat treatment, crystallinity, chemical content, mechanical property

## Abstract

The aim of this study was to investigate the effects of the heat treatment time and initial moisture content of bamboo on the corresponding chemical composition, crystallinity, and mechanical properties after saturated steam heat treatment at 180 °C. The mechanism of saturated steam heat treatment of bamboo was revealed on the micro-level, providing a theoretical basis for the regulation of bamboo properties and the optimization of heat treatment process parameters. XRD patterns of the treated bamboo slices were basically the same. With the increase in the initial moisture content of bamboo, the crystallinity of bamboo increased first and then decreased after treatment. Due to the saturated steam heat treatment, the content of cellulose and lignin in bamboo slices increased while the content of hemicellulose decreased, but the content of cellulose in bamboo with a 40% initial moisture content increased first and then decreased. The shear strength of treated bamboo changed little within 10 min after saturated steam heat treatment, and then decreased rapidly. During the first 20 min with saturated steam heat treatment, the compressive strength, flexural strength, and flexural modulus of elasticity of the treated bamboo increased, and then decreased.

## 1. Introduction

With the rapid development of China’s economy and the improvement in people’s living standards, the demand for green environmental protection materials such as bamboo and wood in home decoration, furniture, and the construction industry is increasing dramatically, which is unlike the current situation where wood resources are relatively scarce and import is becoming more and more difficult in China. Bamboo resources are widely distributed in China, with the largest area, species, and output in the world [1]. At the same time, bamboo has the characteristics of great environmental adaptability, fast growth, light weight, and excellent mechanical properties and so on, which is an important biomass material to replace wood. According to the 9th National Forest Resources Inventory Report (2019), the bamboo forest area in China is 6.41 billion hectares, of which the moso bamboo forest area accounts for ~73%. However, bamboo contains a large number of polysaccharides and starch. This makes it vulnerable to a variety of molds. Therefore, the question becomes how to make good use of bamboo to overcome its mildew, poor dimensional stability, and other shortcomings. Achieving the real realization of “replacing wood with bamboo,” alleviating the pressure of raw material shortage in the wood processing industry, and improving the development level of the bamboo industry in China are the critical issues in the development of the bamboo industry [2,3].

Studies have shown that after short-term pyrolysis of bamboo in a protective gas environment or liquid medium at 160–250 °C, all kinds of chemical components in bamboo will occur, including pyrolysis and polycondensation to a certain extent. While releasing its growth stress and drying stress, it reduces the content of hemicellulose and the hydroxyl in the cell wall of bamboo. This will improve the physical and chemical properties and reduce the hygroscopicity and water absorption, thus improving the dimensional stability and biological durability of bamboo [4,5,6]. At present, high-temperature heat treatment methods mainly include hydrothermal treatment [7], oil heat treatment [8], and gas-phase heat treatment [9,10]. Although the first two methods have been applied in small-scale industrialization all over the world according to their abilities to improve the physical and chemical properties of bamboo, these methods have many drawbacks. For example, a large amount of oil or water will be absorbed during the treatment process. These complex processes make it difficult to process and deal with waste. In addition, the cost of labor and time is very high. Conversely, as one of the gas phase heat treatments, saturated steam heat treatment is supposed as an environmentally friendly and efficient treatment method, which has been proven to improve the physical and chemical properties of bamboo and decrease color deviation at the same time, increasing the corresponding added value [11].

In recent years, more and more scientists have paid attention to saturated steam heat treatments. It is an economical and eco-friendly method to modify bamboo. This method can reduce its hydrophilicity and modify its weather resistance, dimensional stability, and durability. When applied to actual production and application, the mechanical strength, anti-corrosion, and anti-mildew performance of the products will be improved. Investigations prove that saturated steam heat treatment can not only make full use of the original moisture in bamboo to improve heat transfer efficiency, but also effectively improve the physical and mechanical properties and reduce pollution [12,13,14]. At present, there are many studies on the effects of treatment temperature and treatment time on the properties of high-temperature heat-treated bamboo all over the world [15,16,17]. Appropriate temperature can effectively reduce the hygroscopicity and water absorption of bamboo and improve the dimensional stability and durability of bamboo. If the temperature is too high, it will cause surface hardening of the bamboo and reduce its mechanical strength, thus reducing the comprehensive strength of bamboo and affecting its application in bamboo products and the bamboo industry [18,19]. However, there are few research studies on the effect of the initial moisture content of raw materials on the properties of treated bamboo. However, the existence of water in biomass materials not only affects the heat transfer efficiency in the material, but also affects the change law of material chemical composition in the process of heat treatment. For example, some studies have pointed out that the presence of water will aggravate the pyrolysis of hemicellulose, thus affecting the properties of the treated wood [20]. At the same time, bamboo growth conditions, cutting season, and storage time will affect its initial moisture content in the production process. If it is not controlled before high-temperature heat treatment, it will easily produce differences in product performance between different batches. In addition, changes in the chemical composition, crystallinity, and relationship between heat-treatment process parameters in the heat treatment process are rarely studied, which will affect the final product performance of the treated material. Therefore, consider the great effect of moisture content on wood and the need in actual production. We think it is very considerable to study the effect of different initial moisture contents of bamboo on the physical and chemical properties.

In this paper, moso bamboo was taken as the research object and heated using 180 °C saturated steam. The effects of saturated steam heat treatment on the chemical composition, crystallinity, and mechanical properties of bamboo were studied. The mechanism of saturated steam heat treatment of bamboo was revealed on the micro-level, providing a theoretical basis for the regulation of bamboo properties and the optimization of heat-treatment process parameters.

## 2. Results and Discussion

### 2.1. Effect of Saturated Steam Heat Treatment on the Crystallization of Bamboo

**Figure 1:**
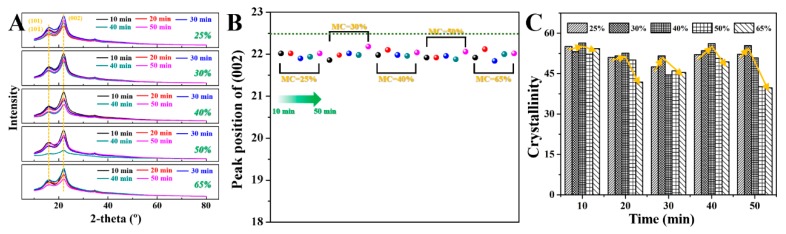
(**A**) The XRD curves of the bamboo slices after 180 °C saturated steam heat treatment. The corresponding diffraction peak location of cellulose (002) plane (**B**), and relative crystallinities (**C**) of bamboo slices with different initial moisture contents and heat treatment times.

Figure 1A shows XRD curves of the bamboo slices after saturated steam heat treatment at 180 °C with separate initial moisture content and heat treatment duration. It can be seen from the figure that the XRD patterns of the bamboo slices after treatment are basically the same and they both show two primary diffraction peaks. The characteristic peak corresponding to the smaller diffraction angle is the dispersion peak produced by the amorphous state, which represents the (101) and (10$\bar{1}$) crystal plane. Figure 1b shows the change in the diffraction peak position of the cellulose (002) crystal surface of bamboo slices with different initial moisture contents after saturated steam heat treatment. From the XRD curves, we can see that the positions of the (002) diffraction peaks are all in the range of 21.84–22.18 °C. It has not changed much. This shows that the saturated steam heat treatment process has no obvious effect on the crystal region of the bamboo. However, unlike wood, the positions of the (002) diffraction peaks of treated bamboo are lower than those of untreated bamboo (22.50 °C). The analysis shows that this is mainly due to the macroscopic residual stress produced in the cellulose of bamboo slices under the action of saturated steam heat treatment, which leads to lattice distortion. As a result, the lattice anisotropy shrinks, the spacing between crystal planes becomes larger, and the corresponding diffraction angle decreases [21].

The results show that the change in crystallinity of cellulose in biomass materials is directly related to its physical and mechanical properties. The crystallinity of cellulose in bamboo slices is generally the percentage of the crystalline region of cellulose in the whole cellulose. It is calculated by the Segal formula.

(1) $$C_{r}=\left(\frac{I_{002}-I_{am}}{I_{002}}\right)\times 100\%\;\;\;\;\;\;\;\;$$

$C_{r}$ is the relative crystallinity (0−1), $I_{002}$ is the maximum intensity of the 002 diffraction peak, and $I_{\mathrm{am}}$ is the non-crystalline diffraction intensity. The result is shown in Figure 1C. It is obvious from the figure that, regardless of the treatment time, the relative crystallinity of the bamboo materials with initial moisture contents of 50% and 65% is low. This is mainly because the saturated steam heat propagates efficiently inside the wet bamboo slices, so a lot of hemicellulose hydrolyzes. At the same time, more acetic acid is generated to catalyze the degradation of cellulose microfibrils. On the other hand, the bamboo with a large initial moisture content evaporates lots of water during saturated steam heat treatment, so the microcrystalline structure of cellulose in bamboo is destroyed to a certain extent. It is noted that, with the increase in the initial moisture content of bamboo, the crystallinity of bamboo increases first and then decreases after treatment. This is mainly due to the improvement in the transmission efficiency of saturated steam heat in bamboo slices with the increase in initial moisture content, thereby promoting the hydrolysis of hemicellulose to produce organic acids. In the acid condition, the cellulose in the non-crystallized area undergoes catalytic degradation first. With the prolongation in heat treatment time, this part of the microfibrils recrystallized. At the same time, with the “bridged reaction” between the hydroxyl groups of the cellulose molecular chains in the undegraded amorphous region of the bamboo slices to form ether bonds, the crystallinity of bamboo slices increases with the initial moisture content.

### 2.2. Effect of Saturated Steam Heat Treatment on Chemical Functional Groups of Bamboo

**Figure 2:**
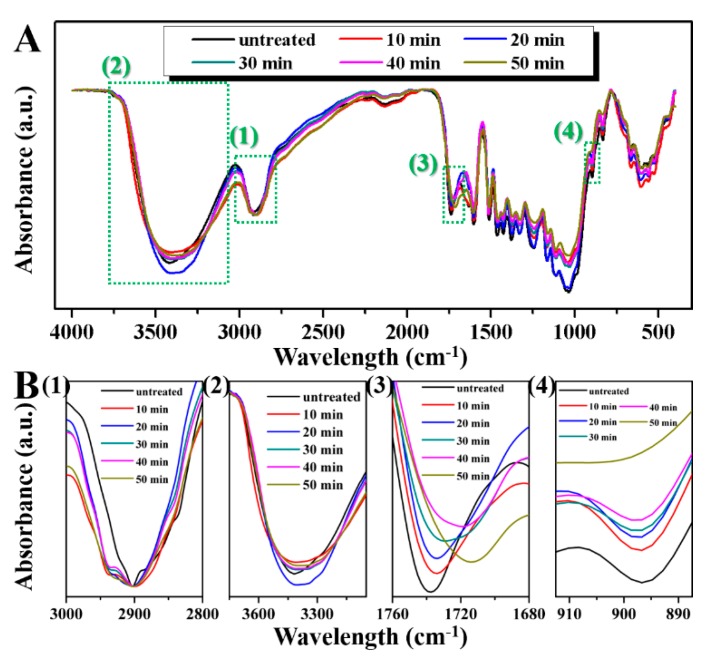
Complete (**A**) and partial (**B**) FTIR curves of the bamboo slices with 25% initial moisture content treated by saturated steam heat at 180 °C with different times.

The FT-IR spectra of the bamboo slices with a 25% initial moisture content treated by saturated steam heat at 180 °C with different times, as shown in Figure 2A, show enlarged FT-IR spectra of different infrared absorption bands. As the C-H functional group of cellulose does not change during heat treatment, we normalize the C-H bending vibration absorption peak at 2900 cm^−1^ (Figure 2B(1)) [22]. It can be seen from the figure that with the prolongation in heat treatment time, the absorption bands of other functional groups of the bamboo slices change significantly. The absorption peaks at 3403.7cm^−1^ belong to the bending vibration peaks in the hydroxyl plane. The intensity of the peak increases and then decreases with the prolongation in heat treatment time. This is mainly because, in the initial stage of the saturated steam heat treatment, the water in the bamboo slices is ionized first by heating and then generates hydrogen ions. Hydrogen ions attack the ether bonds on the hemicellulose molecular chain to cause an electrophilic reaction, thereby promoting the degradation of hemicellulose into D-xylose and 4-O-methyl-D-glucuronic acid, so the content of hydroxyl in the treated bamboo slices increases [23]. However, with the prolongation in heat treatment time, hemicellulose further degrades into smaller molecules and produces acetic acid. The dehydration polycondensation of cellulose hydroxyls catalyzed by acetic acid results in a remarkable reduction in the number of free hydroxyls. The intensity of the characteristic absorption peak decreases. This is the main reason for the increase in dimensional stability and the decrease in the hygroscopicity of biomass materials through high-temperature heat treatment. As shown in Figure 2B (3), the bands at 1680−1760 cm^−1^ belong to the C=O stretching vibration absorption peaks of hemicellulose. The intensity of the peak decreases with the prolongation in heat treatment time. This is attributed to the deacetylation reactions inside the hemicellulose molecules during the heat treatment [24]. However, with the reaction time reaching 50 min, the peak intensity increased slightly. With the progress of the reaction, a certain blue shift gradually appeared in the absorption peak of the C=O stretching vibration. This is mainly due to the esterification of lignin in the bamboo cell wall catalyzed by acid reagent under acidic and high-temperature conditions. It leads to the increase in the content of Ar-O-Ar (AR: Benzene ring) functional groups in bamboo slices. As shown in Figure 2B (4), the vibration peak at 896 cm^−1^ is related to the out-of-plane C-H distortion of cellulose and hemicellulose, as well as the β-glucosidic bond [25]. The intensity of the absorption peak gradually diminishes with the prolongation in heat treatment time. This is mainly due to the pyrolysis of hemicellulose resulting in a large number of glycoside bonds breaking and converting into small molecular compounds and then lost.

### 2.3. Effect of Saturated Steam Heat Treatment on Cellulose, Hemicellulose, and Lignin of Bamboo

**Figure 3:**
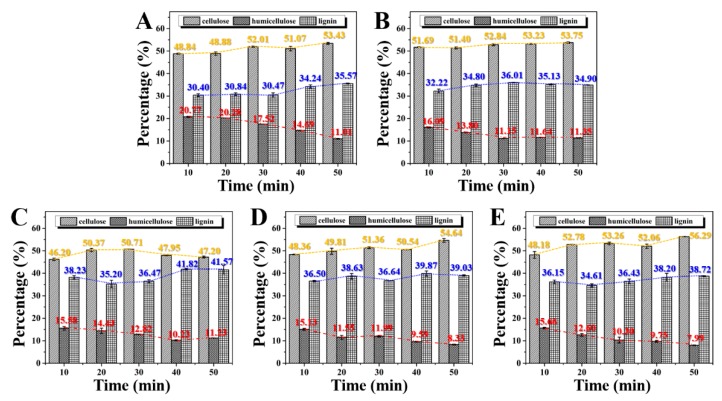
(**A**–**E**) The percentage of lignin, cellulose, and hemicellulose of bamboo slices after the saturated steam heat treatment with different initial moisture content.

Figure 3 shows the percentage of cellulose, hemicellulose, and lignin of bamboo slices after the saturated steam heat treatment with different initial moisture contents. It can be seen from the figure that the hemicellulose content in bamboo slices with different initial water contents after heat treatment decreases with the prolongation in heat treatment time. The contents are 11.01%, 11.35%, 11.23%, 8.33%, and 7.99% after 50 min. This result indicates that saturated steam heat treatment can effectively improve the degradation efficiency of hemicellulose in bamboo and then improve the dimensional stability and antiseptic and antibacterial properties of bamboo products. Besides, with the increase in initial water content, the content of the hemicellulose has a gradual decrease after 50 min. The result shows that the initial moisture content in bamboo has a certain promotion effect on the degradation of hemicellulose. At the same time, with the decrease in hemicellulose content in bamboo slices, the lignin content increases with the prolongation in heat treatment time. Compared to hemicellulose and cellulose, lignin has better hydrophobicity and chemical reaction inertness because of its lower hydroxyl content. As the most heat-resistant component in bamboo, the slight increase in lignin content may be due to the condensation and cross-link reactions of lignin or the production of compounds featuring aromatic ring products induced by heat treatment [26]. It is noted that, with the prolongation in heat treatment time, the content of cellulose and lignin in bamboo slices increases while the content of hemicellulose decreases. However, the content of cellulose in bamboo with a 40% initial moisture content increases first and then decreases. The results show that despite the large amount of acetic acid produced by the thermal degradation of hemicellulose, the degradation of cellulose in the amorphous region can be catalyzed. However, hemicellulose degradation is severe under saturated steam heat treatment at 180 °C. Therefore, the relative content of cellulose increases more with the reaction time. Lignin is esterified under the catalysis of acidic reagents. As a result, the number of hydroxyl groups decreases and the number of carbonyls increases, which is equivalent to replacing the hydroxyl group by the carbonyl with weak hygroscopicity [27,28]. However, after 30 min saturated steam heat treatment, the degradation rate of cellulose in the amorphous area of bamboo slices with 40% initial moisture content is faster than that of hemicellulose, which makes the relative content of cellulose begin to decline. At 40 min, the degradation reaction of hemicellulose is basically completed, and the degradation reaction of cellulose in the amorphous region under the catalysis of acetic acid is still ongoing, resulting in a further reduction in the relative content of cellulose and a slight increase in the relative content of hemicellulose. In addition to the three major elements, there are proteins, inorganic substances, and starch in bamboo. Protein accounts for about 1.5–6.0% in bamboo. It was denatured at 180 °C. The denaturation will only degrade, however; it will not affect the mildew resistance and mechanical properties [29]. For inorganic substances, it is rare in bamboo and does not decrease at 180 °C. In addition, there is about 2.0–6.0% starch in bamboo. Studies have shown that the high-temperature oil heat treatment will reduce the starch content in the bamboo. However, the content of protein, inorganic substance, and starch in bamboo is few, so it has little effect on the physical and chemical properties of bamboo.

### 2.4. Effect of Saturated Steam Heat Treatment on Shear Strength of Bamboo

**Figure 4:**
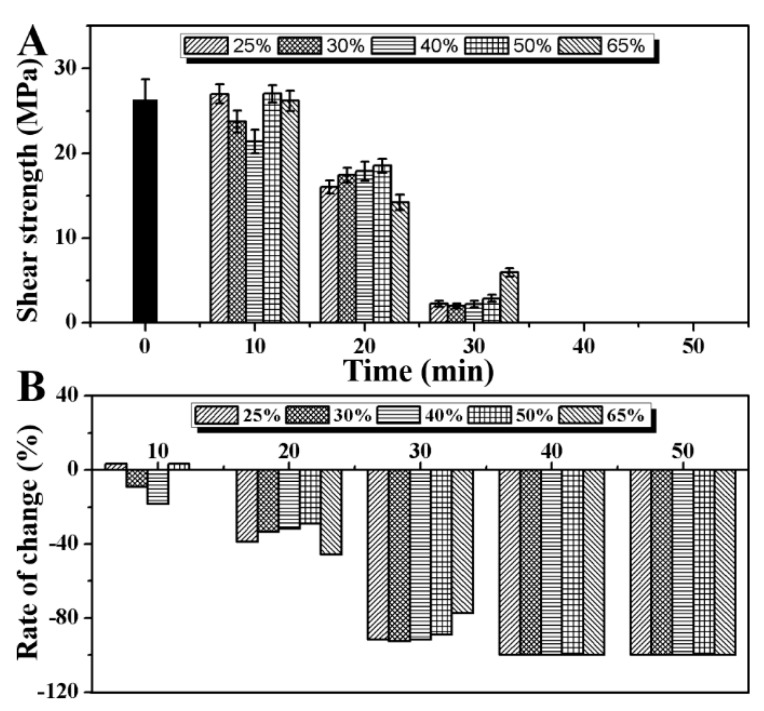
The changes in shear strength (**A**) and corresponding change rate (**B**) with heat treatment time.

Figure 4 shows the change in shear strength of bamboo slices with the increase in heat treatment time before and after saturated steam heat treatment under different initial moisture content conditions. It shows that after 10 min of saturated steam heat treatment, the shear strength of bamboo slices changes little compared to untreated bamboo (26.19 ± 2.60 MPa), the values of which are 26.17 ± 1.23, 27.03 ± 1.01, 21.37 ± 1.41, 23.73 ± 1.30, and 27.00 ± 1.12 MPa. However, with the increase in heat treatment time, the shear strength of bamboo decreases rapidly to about 50% after 20 min. At 30 min, the shear strength of bamboo decreases to less than 5 MPa at different initial moisture contents. This is mainly due to the content of hemicellulose determining the shear strength of bamboo. With the increase in saturated steam heat treatment time, the content of hemicellulose in bamboo decreases gradually (Figure 3). The shear strength of bamboo decreased. In addition, with the continuous reaction of saturated steam heat treatment, the formation of ether linkages between cellulose leads to the decrease in the number of hydrogen bonds. This is one of the reasons why the shear strength of bamboo decreased rapidly after 20 min.

### 2.5. Effect of Saturated Steam Heat Treatment on Compressive Strength Along Grain of Bamboo

**Figure 5:**
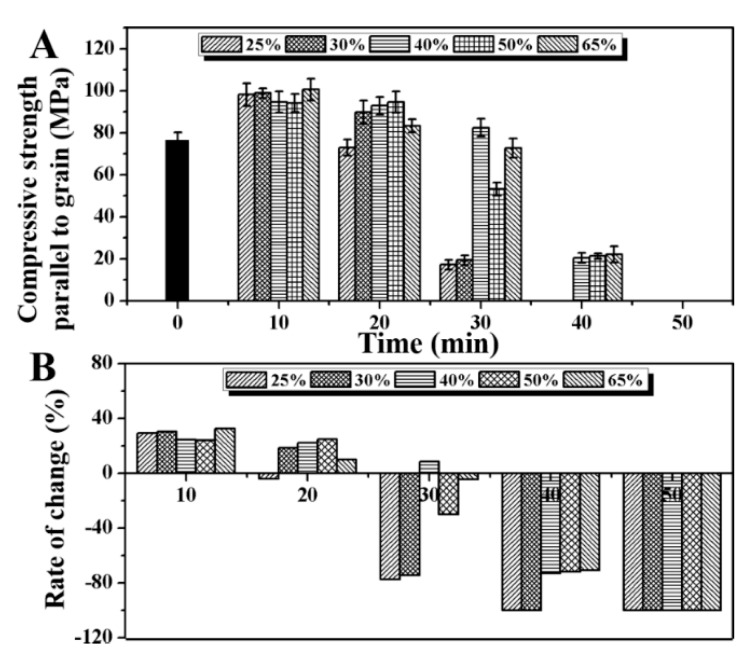
(**A**) Results of compressive strength along grain of bamboo before and after saturated steam heat treatment under different moisture content conditions, and (**B**) the change rate with the prolongation in heat treatment time.

Figure 5 shows the change in the compressive strength along the grain of bamboo after saturated steam heat treatment and its change rate with time. It can be seen from the figure that at the initial stage of the reaction, saturated steam heat treatment promotes the increase in the compressive strength along the grain of bamboo. The change rates of the compressive strength along the grain of bamboo are +29.36%, +30.27%, +24.58%, +24.06%, and +32.53%. After saturated steam heat treatment for 20 min, the compressive strength of the treated bamboo decreases. However, the change in the compressive strength of bamboo with an initial moisture content of 25% is 72.85 ± 3.86 MPa. This is lower than that of untreated bamboo. The compressive strength of the other bamboo is still higher than that of the untreated bamboo, and the change rates are −4.01%, +18.33%, +22.32%, +24.77%, and +9.87%, respectively. In the early stage of saturated steam heat treatment (10–20 min), the compressive strength of bamboo along the grain increases. This is mainly because cellulose plays a supporting role in the three major components of the bamboo cell wall, giving bamboo elasticity and strength. In addition, lignin plays the role of the hard solid material, giving bamboo hardness and rigidity. Both play an active role in the compressive strength of bamboo along the grain. The results of Figure 3 show that the relative content of cellulose and lignin increases gradually during saturated steam heat treatment because the thermal stability of cellulose and lignin is better than that of hemicellulose. As a result, the compressive strength along the grain of bamboo is improved at the initial stage of saturated steam heat treatment [30]. However, with the progress of the reaction, the compressive strength along the grain of bamboo slices with low initial moisture content (25% and 30%) decreases significantly. The change rates at 30 min are −77.30% and −74.50%, and the change rates at 40 min are both −100%. The compressive strength along the grain of the bamboo slices with other initial moisture contents also decreases at 30 min, but the range is not large. It then decreases to 20 MPa in 40 min. The main reason for the decrease in the compressive strength of bamboo along the grain is the shear failure caused by the displacement between the bamboo micelles. Therefore, with the prolongation of saturated steam heat treatment, a large amount of hemicellulose degradation results in the decrease in the shear strength of bamboo (Figure 4), and, finally, the compressive strength along the grain decreases. However, the decrease range of compressive strength along the grain is different among the bamboo with different initial moisture contents. As the water in bamboo is beneficial to improve the steam heat propagation efficiency and promote the formation of the cellulose ether bond, it hinders the generation of intermicellar displacement and inhibits the decline in the compressive strength along the grain of bamboo.

### 2.6. Effect of Saturated Steam Heat Treatment on Bending Strength and Modulus of Elasticity of Bamboo

**Figure 6:**
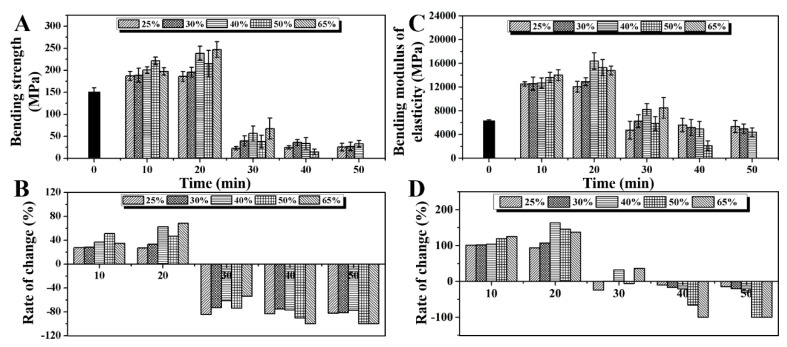
(**A**) Results of bending strength of bamboo before and after saturated steam heat treatment under different moisture content conditions, and (**B**) the change rate with the prolongation in heat treatment time. (**C**) Results of modulus of elasticity of bamboo before and after saturated steam heat treatment under different moisture content conditions, and (**D**) the change rate with the prolongation in heat treatment time.

Figure 6A,B show the bending strength of bamboo after saturated steam heat treatment with the prolongation in heat treatment time and its change rate with time. It can be seen from the figure that in the first 20 min, the bending strength of bamboo shows an upward trend. The change rates of the bending strength of bamboo with an initial moisture content of 25% to 65% after 10 min saturated steam heat treatment are +27.77%, +28.71%, +36.79%, +51.19%, and +34.81%. After 20 min, the change rates of bending strength are +26.95%, +33.49%, +62.96%, +46.87%, and +68.69%, respectively. This phenomenon is basically consistent with the change rule of the compressive strength along the grain of bamboo. The decrease in bending strength of treated bamboo is mainly due to the degradation of hemicellulose interpenetrating between microfibrils and bonding with lignin during saturated steam heat treatment, so the bamboo becomes brittle and the bending strength decreases.

Figure 6C,D show the modulus of elasticity of bamboo after saturated steam heat treatment with the prolongation in heat treatment time and its change rate with time. It can be seen from the figure that, after 10 min, due to the effect of saturated steam heat treatment, the modulus of elasticity of bamboo slices with initial moisture contents of 25% to 65% increases. The rates of change are +101.03%, +101.94%, +103.62%, +118.74%, and +125.11%. After 20 min, the modulus of elasticity of the treated bamboo slices was further increased. In the early stage of saturated steam heat treatment, the increase in the modulus of elasticity of bamboo slices was mainly due to the loss of water in the amorphous area of cellulose. The closer arrangement of cellulose molecular chains and the formation of ether bonds between hydroxyl groups of molecular chains increase the crystallinity of cellulose molecules, thus improving the mechanical properties of bamboo. In addition, due to the degradation of hemicellulose, the relative content of cellulose and lignin increases, which will also lead to the improvement in mechanical properties of bamboo. At the same time, the change rate of the modulus of elasticity increases with the initial moisture content of the bamboo slices. This is mainly because the presence of moisture can not only promote the formation of cellulose ether between the hydroxyl groups in acidic environments, but also promote the degradation of hemicellulose. However, with the prolongation in heat treatment time, the modulus of elasticity of the treated bamboo slices gradually decreases after 30 min, which is mainly due to the large amount of hemicellulose degradation. The acetic acid produced by the degradation of hemicellulose further catalyzes the degradation of cellulose, reducing the cellulose crystallinity (Figure 1C). At the same time, the connection between hemicellulose, lignin, and cellulose is broken and the intercellular layer is split, thereby reducing the mechanical properties of bamboo.

## 3. Materials and Methods

### 3.1. Materials

Natural 6 year-old Moso bamboo (Phyllostachys pubescens L.) was taken from Qingyuan County, Lishui City, Zhejiang Province in China. After cutting out a 2.0 m section of bamboo at a joint with a height of about 1.0 m, the section contains 6 bamboo joints or so. After that, the bamboo tube was divided into 10 slices and each long piece was cut into 6 shorter slices according to the bamboo joints. In the process of truncation, the bamboo segments were removed and further processed. Finally, standard bamboo slices with an average length of 280.0 mm, an average width of 32.0 mm, and an average thickness of 11.6 mm were obtained for use.

### 3.2. Methods

The bamboo slices were divided into 26 groups, with 20 slices in each group. Among them, one group was used as the control group and the other 25 groups were treated in saturated steam heat treatment equipment with different time and initial moisture content conditions. Bamboo slices have been protected by steam in the process of heat treatment. The temperature of heat treatment was 180 °C, and the durations of heat treatment were 10, 20, 30, 40, and 50 min. The initial moisture contents of bamboo slices were 25%, 30%, 40%, 50%, and 65%. Raw bamboo slivers’ moisture contents were roughly adjusted using a drying kiln at 45 °C to obtain bamboo slivers with initial moisture contents (absolute moisture contents) of about 25%, 30%, 40%, 50%, and 65%. Then, the bamboo slivers adjusted accurately by a constant temperature and humidity box and the bamboo slivers with the five moisture contents above were obtained.

### 3.3. Characterization

#### 3.3.1. Fourier-Transform Infrared Spectroscopy (FT-IR) Analyses

IR spectra were recorded by FT-IR (Thermo Scientific Nicolet 6700 FTIR spectrometer, Thermo Fisher Scientific Company, Waltham, Boston, Ma, USA), and each specimen powder together with KBr was pressed to form a tablet before the detection.

#### 3.3.2. X-ray Diffraction (XRD) Analyses

XRD patterns were obtained on a Bruker D8 Advance powder X-ray diffractometer (Bruker, Karlsruhe, Baden wuertenburg, Germany) operated at 40 kV and 40 mA using Cu-Ka radiation (λ = 1.54 Å) with 200 mg of each specimen powder. The data were collected with a 2θ scanning range from 10° to 80°.

#### 3.3.3. Three Major Elements Analyses

The content of three major elements (cellulose, hemicellulose, and lignin) in treated bamboo was determined by National Renewable Energy Laboratory (NREL) method. For details, the concentration of monosaccharides in the supernatants were determined using a high performance liquid chromatography (HPLC) system (LC-2800, Agilent Technologies Inc., Palo Alto, California, USA) with a refractive index detector, and 5 mM H_2_SO_4_ solution was used as the eluent at a flow rate of 0.6 mL/min.

#### 3.3.4. Testing Methods for Physical and Mechanical Properties

The compressive strength, flexural strength, flexural elastic modulus, and flexural shear strength of treated wood were carried out according to the standard GB/T 15780-1995 (Testing methods for physical and mechanical properties of bamboo). At the same moisture content, the mechanical properties of treated and untreated materials were compared by the following formula:

(2) $$B=\frac{M_{c}-M_{s}}{M_{s}}\times 100\%$$

B represents the change rate of mechanical properties of the treated material, and M represents various mechanical properties. Subscripts C and S represent the treated material and untreated material, respectively. The result of the calculation is positive, which means that the performance of treated wood is better than that of untreated wood and vice versa.

## 4. Conclusions

During the saturated steam heat treatment process, the heat treatment time and initial moisture content have a great impact on the physical, chemical, and mechanical properties of the treated bamboo. With the increase in the initial moisture content of bamboo, the crystallinity of bamboo increases first and then decreases after treatment. Among the treated materials, the relative crystallinity of bamboo with the initial moisture content of 40% is the highest. With the degradation of hemicellulose and cellulose non-crystalline areas and the formation of cellulose ether between the hydroxyl groups, the hydroxyl content of bamboo-treated materials increases first and then gradually decreases. The shear strength of treated bamboo changes little within 10 min after saturated steam heat treatment and then decreases rapidly. During the first 20 min with saturated steam heat treatment, the compressive strength, flexural strength, and flexural modulus of elasticity of the treated bamboo increase. Among the samples, the compressive strength and flexural strength of the bamboo with a 65% initial moisture content reach the maximum after saturated steam heat treatment for 10 and 20 min, at 100.58 and 197.37 MPa. The modulus of elasticity of the bamboo with a 65% initial moisture content reaches the maximum after saturated steam heat treatment for 20 min at 16,373.33 MPa. The decrease in the compressive strength along the grain of bamboo with a lower initial moisture content (25% and 30%) is obviously affected by the saturated steam heat treatment time, and the decrease in the flexural strength and modulus of elasticity of bamboo with a high initial moisture content (50% and 65%) is obviously affected by the saturated steam heat treatment time.
